# Peptidomimetics Therapeutics for Retinal Disease

**DOI:** 10.3390/biom11030339

**Published:** 2021-02-24

**Authors:** Dylan E. Parsons, Soo Hyeon Lee, Young Joo Sun, Gabriel Velez, Alexander G. Bassuk, Mark Smith, Vinit B. Mahajan

**Affiliations:** 1 Molecular Surgery Laboratory, Department of Ophthalmology, Byers Eye Institute, Stanford University, Palo Alto, CA 94304, USA; parsonsd@stanford.edu (D.E.P.); soohyeon@stanford.edu (S.H.L.); youngsun@stanford.edu (Y.J.S.); gvelz@stanford.edu (G.V.); 2 Stanford ChEM-H Medicinal Chemistry Knowledge Center, Stanford University, Palo Alto, CA 94305, USA; mxsmith@stanford.edu; 3 Medical Scientist Training Program, University of Iowa, Iowa City, IA 52242, USA; 4 Department of Pediatrics, University of Iowa, Iowa City, IA 52242, USA; alexander-bassuk@uiowa.edu; 5 Veterans Affairs Palo Alto Health Care System, Palo Alto, CA 94304, USA

**Keywords:** peptidomimetics, retina, drug delivery, formulation, pharmacokinetics

## Abstract

Ocular disorders originating in the retina can result in a partial or total loss of vision, making drug delivery to the retina of vital importance. However, effectively delivering drugs to the retina remains a challenge for ophthalmologists due to various anatomical and physicochemical barriers in the eye. This review introduces diverse administration routes and the accordant pharmacokinetic profiles of ocular drugs to aid in the development of safe and efficient drug delivery systems to the retina with a focus on peptidomimetics as a growing class of retinal drugs, which have great therapeutic potential and a high degree of specificity. We also discuss the pharmacokinetic profiles of small molecule drugs due to their structural similarity to small peptidomimetics. Lastly, various formulation strategies are suggested to overcome pharmacokinetic hurdles such as solubility, retention time, enzymatic degradation, tissue targeting, and membrane permeability. This knowledge can be used to help design ocular delivery platforms for peptidomimetics, not only for the treatment of various retinal diseases, but also for the selection of potential peptidomimetic drug targets.

## 1. Introduction

The delivery of drugs to the eye and their subsequent pharmacokinetics are of great importance in ophthalmology for the treatment of a variety of diseases that can lead to impaired vision or complete blindness. Proteomic studies of the eye have revealed many unique protein targets associated with age-related macular degeneration (AMD), diabetic retinopathy (DR), and a variety of other eye diseases, including inherited eye diseases such as neovascular inflammatory vitreoretinopathy (NIV) [1]. Along with the identification of many new target molecules, the development of potent therapeutics must be accompanied by efficient delivery methods [2]. Effectively engineering and delivering drugs to diseased eye tissues is a crucial step in developing successful therapies. To ensure optimal therapeutic effects, drug candidates must be optimized for the eye, which represents a unique target organ that possesses its own distinct pharmacokinetic properties.

Ocular diseases can be treated by different types of drugs, including small compounds, peptides, proteins, oligonucleotides, and genes. Although small compounds demonstrate excellent therapeutic effects in many clinical settings, their low target-specificities may lead to systemic adverse effects, non-druggability to the particular target molecules, prevalent drug–drug interactions, or poor water solubility [3]. On the contrary, the mechanism of biopharmaceuticals, such as peptides, proteins, and genes, is highly specific and they are generally biodegradable and biocompatible, overcoming many of the drawbacks of small chemical compounds. However, the low membrane permeability of biopharmaceuticals due to their large size and charge, as well as their immunogenicity and enzymatic lability, often limit their practical use. If the therapeutic target is intracellular, these limitations become even more critical.

In recent years, peptidomimetics have gained traction as potential therapeutics, and the number of peptide-based drugs is expected to rise [4,5]. They often exhibit superior selectivity (i.e., low off-target effects) relative to small molecules and are relatively straightforward to synthesize compared to other classes of biopharmaceutics. Peptidomimetics consist of a large class of molecules that mimic the natural protein interactions of peptides [6]. They can possess a high degree of peptide character, where side chain and/or backbone modifications are used to enhance the physicochemical properties while maintaining the general peptide motif. They can also be more small molecule-like, where a non-peptide based chemical scaffold projects substituents in an appropriate orientation to imitate the natural protein–peptide interactions. These possess many of the same advantages of typical small molecule drugs. Based on databases of proteomic studies and the ever-expanding field of structural biology, peptidomimetics can be developed as new drugs to cover a wide range of protein targets. The unique molecular characteristics of peptidomimetics, such as small size and target specificity, makes them promising candidates for use in treating ocular disorders.

Drug delivery with minimal adverse effects and maximal efficacy to the target tissue is one of the biggest challenges faced during drug development. The eye is well compartmentalized, and the drug molecules can be selectively diffused through the distinct segments with different efficiencies. In addition, the eye exhibits moderate accessibility for parenteral drug treatment and even surgical procedures. Due to the structural and metabolic properties in the eye, different methods of administration (i.e., systemic, topical, periocular, intravitreal, suprachoroidal, subretinal injections, etc.) can result in vastly different concentrations of drug delivered to the site of action, often making it a challenge to be within the therapeutic window. The drug properties (i.e., size, charge, hydrophilicity, etc.) also drastically affect the efficacy of administration and the best subsequent path. Therefore, the most suitable delivery route can be adopted depending on the drug properties and the desired site of action. For example, the gold standard for protein drug delivery to retina is through intravitreal injections. Various anti-VEGF (Vascular Endothelial Growth Factor) antibodies are intravitreally administered monthly or bimonthly to patients suffering from the neovascular (wet) form of age-related macular degeneration (wet AMD) [7,8].

This review aims to discuss the potential of small molecule peptidomimetics as promising retinal drugs with their pharmacokinetic consideration. Firstly, we address the various methods for administration of ocular drugs and their subsequent ocular pharmacokinetics, while focusing on delivery to the retina. Considering the fact that molecular weight plays a critical role in determining ocular pharmacokinetics [8], small peptidomimetics exhibit similar pharmacokinetic behaviors to small molecule drugs in accordance with similar molecular size, and so we survey the current literature detailing individual studies on the pharmacokinetics of both peptidomimetics and small molecule drugs to highlight the unique challenges associated with drugging the eye. Next, we discuss various ocular drug formulation strategies to overcome delivery hurdles of peptidomimetic drugs and enhance their bioavailability in the retina.

## 2. Retinal Drug Delivery

### 2.1. Physiology and Delivery Barriers in the Eye

**Figure 1:**
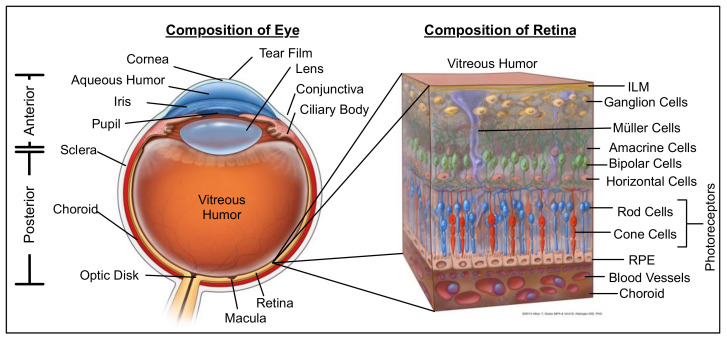
Structure and components of the eye and retina. The eye is composed of various tissues and the retina is located on the inner layer of eyes with distinct cell layers. ILM: internal limiting membrane; RPE: retinal pigment epithelium.

The eye can be divided into two main compartments, the anterior segment containing the cornea, aqueous humor, iris, ciliary body and lens, and the posterior segment containing the vitreous humor, retina, retinal pigmented epithelium (RPE), choroid, and optic nerve (Figure 1). In the anterior segment, the tear film protects the eye against physical damage and infection and is the outermost barrier to the absorption of topically administered drugs [9]. Various proteins in tear film, such as globulin, lysozyme, and albumin, can also affect drug bioavailability. The cornea is composed of the epithelium, hydrophilic stroma, and endothelium [10]. The corneal epithelium barrier exhibits tight junctions to prevent large molecules from penetrating across its membrane. Lipophilicity is another key factor determining whether a drug can permeate this barrier and eventually reach the anterior chamber. The sclera is the opaque, white-colored connective tissue that covers the outer part of the whole eyeball, except for the cornea. It maintains the shape of the eye and has a relatively high permeability. The conjunctiva is a thin, transparent mucus layer lining the inside of the eyelid that covers the sclera. It is highly permeable, and drugs can rapidly travel to the blood/lymphatic system through conjunctival capillaries. The aqueous humor is the fluid filling the anterior chamber [11,12]. Since most tissues in the anterior segment do not contain blood vessels, the aqueous transports nutrients and oxygen. It can influence intraocular pressure, which when mis-regulated can lead to the onset of glaucoma. The lens, ciliary body, iris, and pupil play important roles in visual function. For example, the ciliary body, located at the edge of the lens, can change the lens shape in order to control the visual acuity. However, the ciliary body, with its cellular layers and fenestrated capillaries, is only recently being considered critical to ocular drug delivery. The posterior segment contains the vitreous body, the largest chamber in the eye. This chamber is filled with vitreous humor, a gel-like fluid composed of 98–99% water [13]. This fluid supports the lens and maintains the shape of the vitreous chamber, which is crucial for light to pass to the retina. The vitreous body contains hyaluronic acid, glucose, ions, collagen and we found over 2000 nonredundant proteins [14,15], due to the presence of hyaluronic acid, the vitreous possesses net anionic character. Proteins in the vitreous consist of structural proteins such as collagen, fibrillin and cartilage oligomeric protein, as well as non-structural proteins such as albumin [16,17]. These non-structural proteins may play an important role in a variety of ocular diseases as elevated protein concentrations have been shown in patients with diabetic retinopathy [18]. In addition, the vitreous contains a significant fraction of intracellular proteins released from other tissues in the posterior chamber [19,20]. These proteins (e.g., proteases, esterases, etc.) have the potential to interact with molecules introduced via intraocular injection. The retina is the innermost component and the photographic layer of the posterior segment (Figure 1) [21]. It is composed of an internal limiting membrane (ILM), a neural retina, and the retinal pigment epithelium (RPE) [22]. The neural retina contains optic nerve fibers and a variety of cells, such as retinal ganglion cells, Müller cells, amacrine cells, horizontal cells, retinal capillary endothelial cells, and photoreceptor cells. The RPE is a polarized monolayer of pigmented cells with tight junctions. The choroid, located between the retina and sclera, is a highly vascularized layer [23]. Since the retina continuously processes signals from light, this is a high energy demanding tissue. Retinal capillary endothelial cells and choroidal blood vessels supply oxygen and nutrients to the inner and outer retina, respectively.

### 2.2. Delivery Route and Pharmacokinetics of Various Retinal Drugs

For the treatment of ocular disease, the ideal delivery route can be selected based on the physicochemical properties of drug molecules, target tissue proximity, ocular pharmacokinetics and patient convenience. In this section, we describe various administration routes of ocular therapeutics to target the retina and their pharmacokinetic barriers.

#### 2.2.1. Systemic Delivery

**Figure 2:**
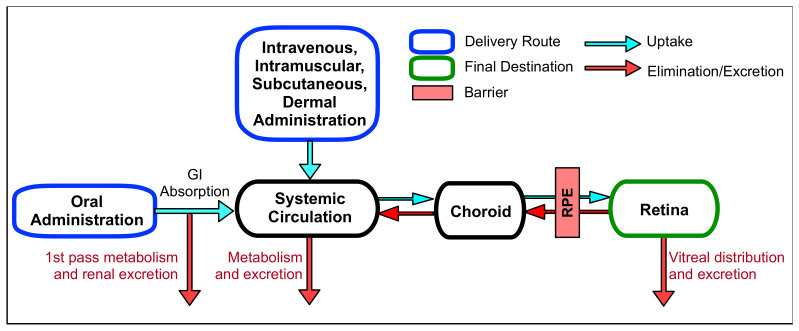
Systemic drug delivery to the retina via oral, intravenous, intramuscular, subcutaneous, and dermal administration. The drugs administered via these routes need to be intact against metabolism and excretion in gastrointestinal (GI) tract and systemic circulation.

Systemic delivery is accomplished through oral administration in the form of a tablet or liquid consumable or through parenteral routes, such as intravenous, intramuscular, subcutaneous, and intradermal administration in an injectable form (Figure 2). Due to low ocular bioavailability and high systemic exposure, these methods are atypical for drug delivery in ophthalmology. To reach the retina, systemically administered drugs need to penetrate the blood ocular barriers, composed of the blood aqueous barrier (BAB) and the blood retinal barrier (BRB). These barriers are important to prevent the entry of toxic molecules and pathogens into the eye and maintain ocular homeostasis. The BAB is built by the tight junctions of nonpigmented epithelium in the ciliary body, iris epithelium, and iris endothelium. These tight junctions play a key role in maintaining the chemical composition of the aqueous humor. The BRB, consisting of the endothelia of the retinal capillaries and RPE, drastically limits the delivery of drugs from the systemic circulation to the retina due to its tight junctions. Without active transport, drug molecules rely on passive diffusion across these membranes. The tight junctions of the BRB have been shown to exclude molecules larger than 2 nm in size [24]. The permeation of small molecules depends largely on the lipophilicity of said compound. Systemic metabolism is another hurdle for systemic drug administration [25]. Before the drug can reach the eye, it must first pass-through systemic metabolism and be exposed to hepatic enzymes within the liver. This process greatly reduces the concentration of drug that can reach the retina.

A previous pharmacokinetic study of the steroid dexamethasone, commonly used to treat both anterior and posterior ocular diseases, compared the pharmacokinetics of intravitreal, intravenous, and subconjunctival delivery by measuring plasma levels over 24 h. The study revealed that after intravenous injection, plasma concentrations were rapidly depleted, meaning the majority of the drug was quickly cleared from the system, making absorption into ocular tissues unlikely. Intravitreal injection showed a depot effect, slowly releasing the drug into the systemic circulation [26]. Fast-systemic clearance was also true for subconjunctival administration. Unfortunately, the authors did not measure the effective concentrations in ocular tissues to compare the ocular distribution for these methods of administration.

Systemic toxicity is also a critical drawback for systemic drug delivery. High dose regimens are often needed to overcome poor drug permeability across the BRB, and systemic metabolism has the potential to exert a toxic effect in non-targeted organs. For example, adverse systemic effects such as bradycardia, arrythmia and asthma have been observed with the oral administration of beta-blockers for the treatment of glaucoma [27]. In addition, sorbinil, a small molecule aldose reductase inhibitor for the treatment of diabetic retinopathy, failed in late clinical trials due to systemic toxicities and poor pharmacokinetics [28]. In addition, since the repetitive systemic exposure of drugs can trigger immune responses, potential immunogenicity of drug and additives should be considered. It goes without saying that any adverse side effect should be avoided when possible, because side effects can decrease a patient’s quality of life and undermine continued treatment.

Although ubiquitous use of oral drugs for eye disease treatment has proven quite challenging to demonstrate in vivo, small molecule drugs do possess the ability to permeate the blood ocular barriers and therefore can potentially be administered through systemic delivery. For example, orally administered acetazolamide, a carbonic anhydrase inhibitor, effectively decreases intraocular pressure (IOP) [29]. Certain steroids are successfully administered orally for the treatment of Graves’ ophthalmopathy, to reduce overall inflammation [30]. The oral antibiotic moxifloxacin is used for ocular infections [31].

#### 2.2.2. Intraocular Delivery

**Figure 3:**
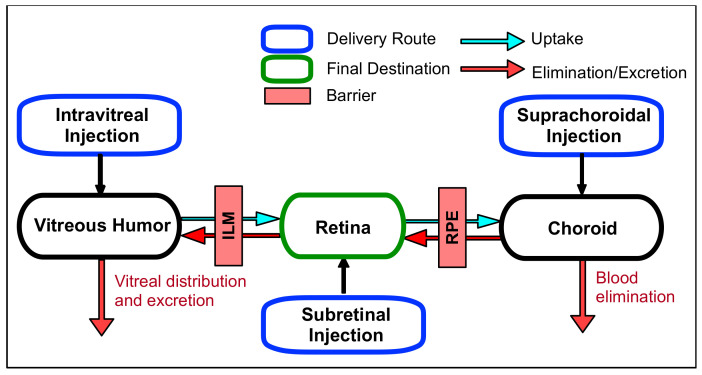
Intraocular injection delivering drugs in close proximity to the retina. After intravitreal or suprachoroidal injection, the only major barrier present is the internal limiting membrane (ILM) and retinal pigment epithelium (RPE), respectively. Subretinal injection directly delivers the drug to the retina.

Intraocular administration is the injection or implantation of a sterile solution or device within the eye via intravitreal, subretinal, or suprachoroidal delivery routes (Figure 3). Among these delivery routes, intravitreal injection is the main drug delivery method to target the retina with higher drug bioavailability by introducing the drug directly to the posterior segment, avoiding several rate-limiting delivery barriers and alleviating many of the complications associated with systemic toxicities. Unlike the RPE, the vitreous has a loose barrier for diffusion with an estimated mesh size in the range of 500 nm [32]. This means that the vitreous is relatively open and allows for movement of large and small molecules as well as nanoparticles via diffusion and convection [33,34]. More important than size for predicting molecular movement in the vitreous is the charge of the species. Neutral and anionic molecules diffuse relatively freely while cationic species are highly restricted due to the electrostatic interactions with the anionic hyaluronic acid polymers in the vitreous [35]. Käsdorf et al. compared the diffusion efficiency of polystyrene nanoparticles with different surface modifications, such as carboxyl-terminated, PEGylated, and amine-terminated. In the vitreous humor from animals, the diffusion rate of positively charged particles was greatly suppressed compared to their neutral or anionic counterparts. In addition to diffusion through the vitreous body, intravitreally administered drugs need to pass through the internal limiting membrane (ILM) to reach the retina. This membrane contains a high density of extracellular matrix (ECM), such as collagen, laminin, and heparin sulphate proteoglycan (HSPG), that affect drug permeability, and its composition shows age-related changes [36]. The drugs with high binding affinities to this ECM can effectively penetrate the ILM to reach the retina. For example, adeno-associated virus of serotype 2 (AAV2) exhibiting a binding affinity to HSPG showed an excellent retinal transduction after intravitreal injection, while other AAV serotypes and modified AAV2 without HSPG-binding capacity failed to transfect [37]. High metabolic activity in the RPE is another critical factor that can reduce drug bioavailability. Intravitreally injected retinal drugs can be degraded in the eye by metabolic enzymes like cytochrome P450 and esterase [38]. PEGylation, an attachment of hydrophilic polyethylene glycol (PEG) polymer to the molecules either covalently or non-covalently, or nanoparticle encapsulation can enhance the stability of enzymatically labile drugs, which will be discussed in subsequent sections. Drug clearance after intravitreal injection occurs posteriorly or toward the anterior segment of the eye depending on the drug size, property, and concentration gradient. Drugs that diffuse toward the posterior segment penetrate the ILM to reach the retina. The following clearance efficiency is dependent on the penetrating efficiency through the RPE tight junction. Small lipophilic compounds can be transported with higher efficacy than large hydrophilic compounds [39]. Once they escape the retinal layer, drugs in the choroid can enter the systemic circulation via choroidal blood vessels. Drugs diffused toward the anterior segment are drained out via the trabecular meshwork and Schlemm’s canal and are finally eliminated via blood or lymphatic vessels.

Subretinal injection is another efficient delivery route to target the retina. Since this administration can deliver drugs directly into the retina, it is the most efficient route for large biopharmaceuticals with low membrane permeability and poor molecular stability. Subretinal injection is the main method for human retinal gene therapy [40]. Drugs delivered via subretinal injection are exposed to the inner layer of the retina where photoreceptor cells, ganglion cells, and neuronal retinal cells are located, but there is limited movement toward the outer layer of the retina due to the tight junction of the RPE. For this reason, injected drugs are mainly cleared via the anterior segment pathway, not through choroidal vessels, despite their proximity. Retinal and RPE damage is the main risk of this administration route [41].

Supra-choroidal injection is a newer method of delivery, introduced in 2002, but has yet to make it into the clinic [42]. With this method, the drug is deposited in the suprachoroidal space, located beneath the sclera, via microneedles or cannulas. Since the site of injection is close to the choroid, this administration route exerts a high drug bioavailability for choroidal disease treatment. Drug movement in suprachoroidal space may be limited by the ciliary arteries of the choroid, resulting in uneven drug distribution [43]. Consequently, the ideal drug injection site corresponds to the target region of the choroid. For retinal delivery, both the drug clearance rate and RPE permeability are crucial factors in determining the bioavailability of supra-choroidally injected drugs. After supra-choroidal injection, a majority of the drug is rapidly lost to systemic circulation due to the high choroidal blood flow, resulting in very poor half-lives. Ranta et al. predicted the retinal bioavailability via a suprachoroidal injection route to be between 0.2–4% using their kinetic models [44]. For small molecule sodium fluorescence (MW = 376 Da), the suprachoroidal half-life was determined to be 1.2 h with total clearance from the eye observed after 24 h [45]. Macromolecules have longer suprachoroidal half-lives. For example, 40 kDa and 250 kDa dextrans have experimental half-lives of 3.6 and 5.6 h, respectively. Interestingly, the antibody Bevacizumab (MW = 149 kDa) has an even greater half-life of 7.9 h. Comparing the half-life of the 250 kDa dextran and Bevacizumab, it is apparent that, besides molecular size, other factors, such as charge, lipophilicity, and flexibility, also play important roles in clearance rates. Contrary to the rapid clearance of small- and macro-molecules, particles have been observed to remain for months. With limited clearance, supra-choroidal injections of drug-releasing implants possess great potential as an effective retinal drug delivery strategy.

#### 2.2.3. Periocular Delivery

**Figure 4:**
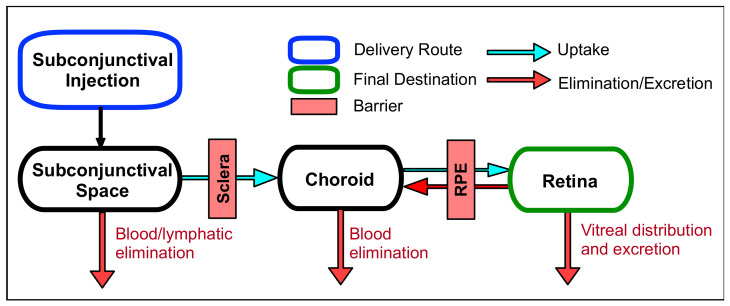
Drug pathway after subconjunctival injection. Drug in the subconjunctival space can reach the retina by passing through sclera and retinal pigment epithelium (RPE) barriers.

Periocular delivery is another method to introduce drugs directly into the eye. This technique involves injecting the drug into one of the spaces surrounding the eye, including the sub-conjunctival, sub-Tenon’s, peribulbar, retrobulbar, and posterior juxtascleral space (Figure 4). This method is less invasive than intraocular injections and lowers the risk of endophthalmitis (intraocular infection) and cataract formation. Of all the injection spaces surrounding the eye, the sub-conjunctival space is the most commonly used in clinical practice. By avoiding the corneal barriers, sub-conjunctival injections result in higher bioavailabilities in the anterior segment compared with topical delivery. Injected drugs can reach the posterior segment via the conjunctival-sclera pathway, but their bioavailabilities are typically much poorer. Retinal bioavailability has been determined to be roughly 0.1%, similar to topically administered drugs but much lower than intravitreal administration [46]. This low bioavailability can be attributed to two main factors: rapid clearance to the systemic circulation via choroidal blood vessels and a multitude of barriers between the retina and sub-conjunctival space. After administration of small molecules, 80–95% is rapidly lost to the systemic circulation. The low bioavailability in the retina restricts the usage of sub-conjunctival injections to compounds that are highly potent. Although more invasive, supra-choroidal delivery circumvents the necessity to permeate the sclera and is a more appropriate method for delivering drugs to treat diseases of the retina compared to sub-conjunctival delivery. Since the typical injection volume of subconjunctival delivery (100–500 microliters) is higher than that of supra-choroidal delivery (50–200 microliters), this administration route can be useful for larger drug injection volumes or controlled drug release [47].

#### 2.2.4. Topical Delivery

Topical delivery, a direct instillation of drugs to the ocular surface, is a very convenient and noninvasive method to deliver drugs to the eye. Due to the proximity of the administration site, it is a preferred drug delivery route to treat anterior segment diseases, such as glaucoma, cataract, dry eye, corneal and conjunctival inflammation and infections [48]. Ninety percent of ophthalmic drugs on the market are eyedrop compositions. Topical dosage drugs include prostaglandin analogues, beta-blockers (timolol), alpha-2 agonists (brimonidine, apraclonidine), and carbonic anhydrase inhibitors (dorzolamide, acetazolamide, methazolamide). Steroids (prednisolone) as well as non-steroidal anti-inflammatory (NSAID) drugs have also been administered topically to reduce inflammation caused by certain conditions.

**Figure 5:**
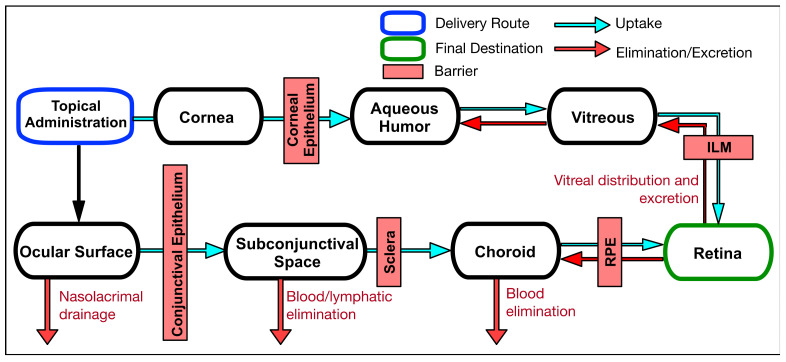
Drug pathway after topical administration. Topical delivery requires the drug to pass through multiple chambers before reaching the retina. This is in conjunction with rapid nasolacrimal drainage from the first compartment which contributes to the poor retinal bioavailability resulting from topical administration.

For retinal delivery, this route of administration is not commonly used because delivering an effective dose to the posterior segment via this administration is extremely difficult. Once topically administered, 95% of drugs are absorbed by the vascularized conjunctiva or nasolacrimal duct and are subsequently eliminated via blood/lymphatic vessels or the gastrointestinal tract, respectively (Figure 5). The remaining drug penetrates the tear film and cornea, which are composed of epithelium, stroma, and endothelium sequentially, to reach the interior of the eye. The drugs in the aqueous humor can be degraded by metabolic enzymes released from the ciliary body. The molecular size, charge, and hydrophilicity of drugs greatly affect the corneal permeability. Since the pore size of the corneal epithelial tight junction is about 2 nm, large or hydrophilic drugs exhibit much lower permeability compared with small and lipophilic drugs. Positively charged molecules can take advantage of this property to bind to the corneal membrane.

There are studies demonstrating significant drug delivery to the posterior segment by topical administration via a trans-corneal route or non-corneal route, such as the conjunctival-sclera pathway [48]. L-timolol, a lipophilic small molecule, can penetrate the corneal membrane and the maximum ocular bioavailability was determined to be 11% [49]. Tang-Lui et al. studied the ocular distribution of small molecule drugs after corneal perfusion and determined that corneal permeability depends strongly on the lipophilicities of drugs [50]. The relative ocular tissue distribution of lipophilic small drugs via the corneal route is as follows; cornea > aqueous humor ≈ iris ≈ ciliary body > anterior sclera > lens. Chien et al. showed that topically administered small lipophilic molecule drugs were delivered intraocularly via the transcorneal pathway, while hydrophilic drugs reached posterior segments via the conjunctival-scleral pathway. Ahmed et al. demonstrated that topically administered large molecule drugs can bypass the corneal epithelium penetration via non-corneal absorption, which is important for the retinal delivery of large biopharmaceutics with poor corneal permeability [51].

To obtain high drug bioavailability after topical delivery, various formulation methods have been developed to enhance penetration through the cornea and prolong retention time in the intraocular space, which are discussed in the following sections.

## 3. Peptidomimetics as Potential Ocular Therapeutics

**Figure 6:**
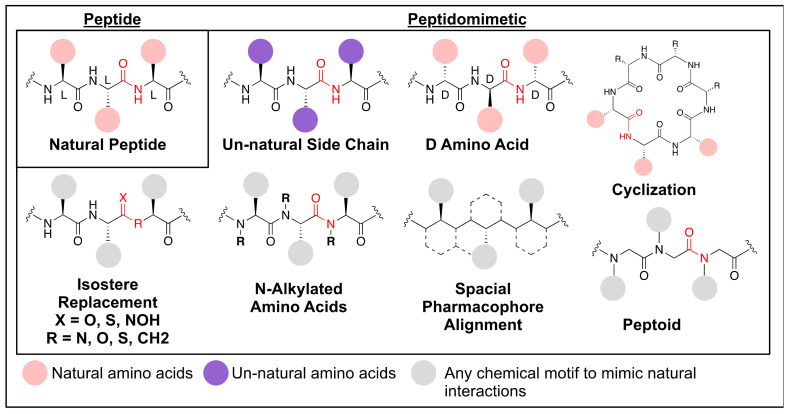
Various synthetic strategies of peptidomimetic. Various peptidomimetics are derived from natural peptide by replacing with un-natural amino acid, or modifying backbone structures to mimic natural peptide functions.

Therapeutic peptides often have certain pharmacophores that interact with target sites on the protein binding pocket and trigger a biological response. A peptidomimetic is a peptide-mimetic molecule containing these pharmacophores, facilitating a similar biological activity to a natural peptide (Figure 6). Based on the structural similarity with peptides, peptidomimetics can be classified into two categories. The first are peptidomimetics that contain the amide linkages that form a peptide backbone. These can have various amino acid side chains, both natural and un-natural, as well as non-amino acid-based moieties on a central peptide-backbone. The second type of peptidomimetics are small molecules which are designed using the biologically active motif, i.e., pharmacophore, of peptides but do not possess an amide backbone [52]. For example, tirofiban, an inhibitor of platelet aggregation, is a non-peptide small molecule mimicking RGD tripeptide (Arg-Gly-Asp) in fibrinogen [53,54].

Using proteomic studies, peptides/peptidomimetics can be designed with a high degree of specificity for the desired target by extrapolating the preferred amino acid residues at the protein sub-sites. This allows you to identify key pharmacophores or protein-ligand interactions. Compared to small molecule drugs, peptidomimetics typically have greater selectivity, which can result in fewer off-target interactions. Compared to proteins, peptides/peptidomimetics exhibit higher physiochemical stability due to their structural simplicity. In addition, they are smaller, have greater activity to mass ratios and better permeability. Peptides/peptidomimetics can be chemically synthesized, allowing for easy modifications, unlike the costly, cell-based production of protein synthesis. The culmination of these attributes makes peptides and peptidomimetics good candidates for therapeutic development. As a result, the number of peptide/peptidomimetic therapeutics has steadily increased over time with several successful therapeutics developed to date, such as Liraglutide and Bremelanotide [55,56].

### 3.1. General Pharmacokinetics of Peptides and Peptidommimetics

Peptides and peptidomimetics exhibit their unique features, distinguished from ones of proteins and small molecules. They can exhibit properties similar to either proteins or small molecules depending on the size of the peptides/peptidomimetics (protein is a polypeptide containing more than about 50 amino acids). However, they also possess their own pharmacokinetic properties. To deliver the drugs at the site of action within biologically relevant concentrations, they should exhibit an efficient membrane permeability. Membrane permeability of the peptides/peptidomimetics is varied depending on their size and lipophilicity. Small and lipophilic peptides/peptidomimetics can penetrate though membranes with a high efficacy, similar to small molecules. Large and hydrophilic peptides/peptidomimetics exhibit poor permeability similar to proteins [57]. To maximize membrane permeability, it is advantageous for peptides/peptidomimetics to be below 700 Da and possess a degree of lipophilicity [58,59].

Peptides are highly susceptible to proteolytic cleavage, which is a crucial challenge associated with using peptides as therapeutics [60]. This cleavage can occur within the majority of tissues due to proteolytic enzymes being expressed ubiquitously throughout the body. This is in stark contrast to the breakdown of small molecules which is typically localized to the liver. This rapid proteolytic cleavage results in very low half-lives for peptides. Peptidomimetic drugs, such as D-form peptides or peptoids, have replaced natural peptides to overcome their short half-lives due to proteolytic cleavage. D-form peptides are synthesized by the replacement of a natural amino acid with its unnatural enantiomer, a switch from the L-amino acid to the D-amino acid. This chirality switch makes the peptide chain more resistant to enzymatic cleavage as most enzymes are chiral and so preferentially react with L-amino acids. D-form peptides possess nearly identical physicochemical properties to the original L-form peptides. Peptoids are another enzymatically resistant peptidomimetic, where the sidechains of residues are linked to the nitrogen atom instead of to the alpha-carbons of the peptide backbone [61,62]. Small molecule peptidomimetics lacking an amide backbone are often more enzymatically stable.

As peptides have become more popular options for different therapies, the unique pharmacokinetics of different peptide administration routes have been explored. Parenteral injection, particularly intravenous, is one of the most common methods to administer peptide therapeutics as it avoids first pass metabolism from liver and gastrointestinal enzymes and results in effectively 100% systemic bioavailability. Subcutaneous and intramuscular administered peptides range in their bioavailability (20–100%) but are still employed in some cases for the purpose of slow and sustained drug release to the systemic circulation [63]. For non-invasive oral administration of peptides, physiological and chemical barriers lead to poor bioavailability, typical between 1–2% [64]. These barriers include the poor absorption of intact peptides across the gastrointestinal membranes and the harsh acidic gastrointestinal environment containing a multitude of proteolytic enzymes. Currently, very few peptide drugs are administered orally and often require extensive formulation to enhance bioavailability or stability. One successful example is cyclosporine A, a cyclic peptidomimetic which can experience up to 30% bioavailability after oral administration [65]. This high oral bioavailability is due to enhanced intestinal absorption as well as enzymatic resistance owing to the cyclic structure of the cyclosporin A. Buccal, intranasal, and inhalation administration techniques can take advantage of highly vascular membranes to deliver the peptide therapeutic in bypassing hepatic and GI metabolism. Although these less-invasive routes result in extremely low bioavailability, they are typically preferred and result in better patient compliance. Of these methods, only 3 intranasal peptide drugs are currently available on the market, desmopressin synthetic analogue, nafarelin, and calcitonin whose molecular weights are below 3500 Da. Their total bioavailability is observed to be 3% [66].

Excretion of therapeutic peptides is accomplished by the same mechanism as the excretion of endogenous peptides, typically proteolytic cleavage down to individual amino acid residues. Locations with high concentrations of proteolytic enzymes, such as the liver, kidneys, small intestine, and blood, are likely important sites where a majority of degradation occurs. Enzymatically resistant peptidomimetics can be secreted intact via the kidney or liver, depending on their lipophilicity [67].

Certain peptides and peptidomimetics have the ability to elicit a potentially problematic immune response. This is atypical for small peptides but has been observed for peptides of molecular weights higher than 4000 Da. Even a low occurrence of an immune response (< 1%) can result in the therapeutic peptide failing clinical trials. For example, taspoglutide, a glucagon-like peptide-1 agonist developed for the treatment of type 2 diabetes, proved effective in phase 3 clinical trials in controlling blood glucose levels but failed to move forward due to systemic reactions.

### 3.2. Ocular Pharmacokinetic for Peptidomimetics

With the ever-growing interest of peptidomimetics for ocular therapeutics, their pharmacokinetics in the eye must be well understood. A better understanding could guide peptidomimetic design towards the production of more potent and effective drugs. While much work has been accomplished separately in both the fields of ocular pharmacokinetics (presented earlier) and general pharmacokinetics of peptidomimetic in a systemic and cellular level, little work bridging the two exists. While many inferences can be made from the existing data on small molecules and antibody drugs, the ocular pharmacokinetics of peptidomimetics warrant exploration to fully understand their unique potential.

Peptidomimetics administered to the eye face several pharmacokinetic challenges, such as membrane permeation, elimination, binding with ocular biomolecules, and enzymatic degradation, which determine their ocular distribution. Membrane permeation and elimination of peptidomimetics in the eyes is likely dependent on their size, charge, and hydrophilicity. Small and lipophilic peptidomimetics can readily penetrate the ocular membranes, such as the RPE, ILM, and outer limiting membranes (OLM), resulting in their efficient ocular distribution. However, high membrane permeability often causes the rapid elimination of drugs. In comparison, large and hydrophilic peptidomimetics have a very limited ocular distribution at the injection site due to their poor membrane permeability, but their retention time is relatively long. A positive charge on the peptidomimetics facilitates efficient penetration through the negatively charged ocular membranes via charge interaction, although the possibility of undesired drug entrapment in negatively charged vitreous polymer networks should be considered. Enzymatic degradation is typically a concern for peptide-derived peptidomimetics although the levels of proteolytic enzymes the drug is exposed to is much lower in the eye compared to the systemic circulation [68]. Proteolytic degradation within the eye can play an important role where levels of proteolytic enzymes are elevated, such as in an aged vitreous. Several hydrolytic enzymes are also active within the retina along with a variety of esterases [69,70]. Protein binding within the vitreous is considered to play a minor role in the pharmacokinetics of most ocular drugs as the protein concentration is significantly lower than in the plasma (0.5–1.5 mg/mL in vitreous compared to 60–80 mg/mL in plasma) [71,72]. While few studies have been conducted, reports on antibiotics show 90–99% unbound fraction [73,74]. However, specific binding to receptors or transporters on ocular membranes can be used to enhance drug delivery, which is discussed further in Section 3.3.4. 

Currently, only a handful of peptidomimetic drugs have been investigated for the treatment of ocular disorders. Suppressors of cytokine signaling 1 (SOCS1)-derived peptidomimetics (a kinase inhibitory region of SOCS1 combined with a cell permeable residue) (15 amino acid residues) and Vasotide (a small cyclic retro-inverted peptidomimetic to target the retinal VEGF receptors) (5 amino acid residues) have been formulated into eyedrops for the treatment of retinal neuroinflammation and neovascularization, respectively [75,76]. Topical administration (2 weeks) of SOCS1 peptidomimetic resulted in significantly reduced glial activation and neural apoptosis induced by diabetes, as well as reduced retinal levels of proinflammatory cytokines in a diabetic mouse model. Vasotide was shown to significantly reduce retinal angiogenesis in a genetically modified mouse model of AMD, an oxygen-induced mouse model of retinopathy, and a laser-induced monkey model of wet AMD. These studies suggest that treatment of certain posterior segment diseases with peptidomimetics via a non-invasive administration method, such as eyedrops, is quite feasible. The N-Acetylcarnosine, a natural histidine-containing dipeptide, was also shown to be effective in an eyedrop formulation for the treatment of glare associated with cataracts [77]. Unfortunately, none of these studies provided any pharmacokinetic parameters for these compounds after administration.

Effective modeling of pharmacokinetics and pharmacodynamics allows for more efficient drug development. It can expedite the pre-clinical to clinical translation while providing information useful for the development of the drug delivery system, such as prediction of the required dosing regimen. The establishment of pharmacokinetic modeling is crucial for peptidomimetic ocular drugs where few examples exist in the literature. Pharmacokinetic modeling uses easily measurable physicochemical properties of a molecule to predict the pharmacokinetics and pharmacodynamics. Important ocular pharmacokinetic parameters, such as clearance rate and volume of distribution, can be reliably predicted using simple molecular characteristics [78]. The typically short half-life of small molecules makes these parameters critical to reach therapeutic drug concentrations in the desired tissue. Audren et al. successfully developed the pharmacokinetic-pharmacodynamic model for the intravitreal injection of triamcinolone acetonide, a small molecule drug to treat diabetic diffuse macular edema [79]. The estimated half-life was determined to be 15.4 ± 1.9 days, which correlates well with the pharmacokinetic study on triamcinolone acetonide where its half-life was determined to be 18.6 days [80]. Using non-invasive optical coherence tomography, the authors were able to monitor the disease alleviation by measuring the thickness of the central macula. Unfortunately, not all ocular diseases possess an easily measurable pharmacodynamic parameter (i.e., macular thickness) and so using this method for prediction is not universal. However, this highlights the feasibility of using pharmacokinetic-pharmacodynamic modeling to predict pharmacokinetic parameters without the need of highly invasive measurements.

### 3.3. Various Drug Formulations and Modified Pharmacokinetics to Target Retina 

**Figure 7:**
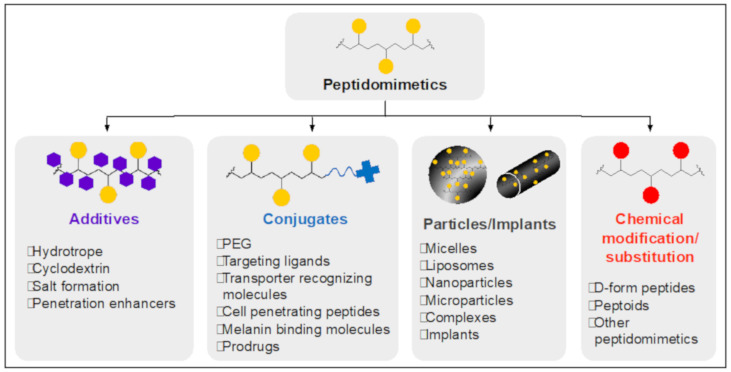
Various formulation strategies of peptidomimetic ocular drugs. For retinal delivery, drugs can be co-administered with additives or formulated by conjugation with functional moieties, encapsulation into particles or implants, and direct chemical modifications/substitutions. Red circle indicates the further modifications in backbone moieties of peptidomimetics.

To enhance the drug pharmacokinetic properties and the consequent therapeutic effects in the retina, ocular peptidomimetic drugs can be chemically or physically modified by using various drug formulation strategies: co-administration, conjugation of functional moieties, particle formation, encapsulation into implant or hydrogel, chemical modification/substitutions, etc (Figure 7) [81]. One or more of these methods can be used to build an efficient drug delivery system with the desired pharmacokinetic properties. Unlike small molecules or short peptidomimetics, large peptides or protein drugs can change their structural properties under certain conditions during formulation, resulting in an undesired decrease in innate drug activity. Therefore, it is necessary to monitor the modified drug activity post formulation. In this section, we introduce various formulations of ocular drugs, including small molecules as surrogates for small peptidomimetic drugs due to the size similarity, to improve their pharmacokinetic properties, such as solubility, retention time, enzymatic resistance, tissue specificity, and membrane permeability. This information will be valuable to design an efficient retinal delivery platform for peptidomimetic drugs.

#### 3.3.1. Solubility Improvements

Drug solutions or suspensions are the most commonly employed ocular drug dosage form due to their convenience of administration and maximized drug bioavailability in physiological fluids. To obtain a suitable solubility, hydrophobic compounds can be solubilized by aids of excipients, modification to an ionized form, chemical conjugation, encapsulation in the supramolecular structure, etc. [82]. The condition of the delivery medium and drug modifications need to be optimized to guarantee the drug activity and stability.

Hydrotropes and cyclodextrins (CD) are the prevalent excipients to bind with hydrophobic small molecule drugs to effectively solubilize them. Hydrotropes are the compound interacting with hydrophobic molecules to solubilize them in aqueous solution. They can be composed of a variety of chemical motifs such as organic acids, ureas, alkaloids, phenolic derivatives, and aromatic cations. They have amphiphilic molecular features; however, their hydrophobic part is too small to be self-assembled, which distinguishes them from micelle-forming surfactants. Although the drug solubilization mechanism by hydrotropes is not fully understood, Booth et al. suggested that intermolecular interaction of drug/hydrotrope and water activity depression can be a possible mechanism [83]. Kim et al. evaluated the solubility enhancement of 13 water-insoluble drugs in which solubility could be enhanced 1000- to 10,000-fold by using N,N-diethylnicotinamide (DENA) and N,N-dimethylbenzamide (DMBA). Refs. [84,85], Hydrotropic agents efficiently and easily solubilize drugs via a simple solid dispersion method; however, the drug-to-excipient ratio is extremely low. For example, 534 mg/mL of DENA is needed to solubilize nifedipine and progesterone (for glaucoma and dry-eye treatment, respectively) up to 5 mg/mL and 7 mg/mL, respectively. Due to the lipophilic nature of the cell membrane, this solubilizing process can decrease the drug permeability [85]. The overall solubility-permeability tradeoff should be considered when determining the optimal amounts of excipients. Cyclodextrin is a supramolecular compound composed of a hydrophilic backbone along with hydrophobic cavities ideal for hydrophobic drug solubilization. For example, hydrophobic carboxyamidotriazole, applied for neovascular ocular disease treatment, was prepared as a solution dosage form at a concentration of 15 mg/mL for intravitreal injection by using 10% ß-hydroxypropylcyclodextrin [86]. After intravitreal injection in rabbit eyes, the formulation begins to rapidly release the free drug to the surrounding tissues, resulting in a half-life of 31 h in the vitreous. Loftsson et al. formulated dexamethasone suspension using gamma-cyclodextrin at the concentration of 30 mg/mL, which was administered topically and intravitreally [87]. The peak concentrations in the vitreous 2 h after topical administration were comparable to concentrations one month after intravitreal injection, indicating that the topical instillation was not as efficient as intravitreal injection to deliver drugs to the posterior segments.

Salt formation of weakly acidic or basic drugs using their counter ions is also an effective solubilizing method wherein the counter ions released in water can build a favorable pH condition for drug solubilization. In the case of topical drug dosage, however, some salts can cause pain and irritation, resulting in fast clearance by activating the production of lacrimal fluids. For example, epinephrine borate results in less irritation compared to the hydrochloride and bitartrate salts of epinephrine, where the released hydrochloride and bitartrate ions significantly decrease the overall pH [88]. Combined with recent iontophoretic technologies, this drug ionization platform does not only enhance the solubility, but also enables the efficient intraocular delivery of various ophthalmologic drugs [89]. In the study of Güngör et al., the dexamethasone phosphate salt form was topically administered to the rabbit eyes and the following transscleral iontophoresis dramatically increased intraocular concentrations of drug [90].

Direct conjugation of hydrophilic polymers, such as PEG (named PEGylation), can be an alternative to increase the drug solubility. Refs. [91,92], This drug modification requires the active pharmaceutical compound contain a chemically reactive site, such as hydroxyl, amine, or carboxyl group. To prevent the reduction of the drug’s intrinsic activity by PEGylation, PEG needs to be conjugated to the drugs via cleavable linkages, enabling drugs to recover their original structure after PEG being cleaved [93]. PEGylation has been widely used to increase the half-life or reduce the immunogenicity of large molecular biopharmaceutics as well as to enhance the small drug solubility [94,95,96].

The formation of particles, such as micelles, liposomes, nanoparticles, micro-suspension, etc., can be a promising drug solubilization method with high colloidal stability. Binding of hydrophobic drugs to amphiphilic polymers, surfactants, or lipids enables them to form supramolecular structures with a submicron-size, mainly via hydrophobic interactions. For the retinal delivery of cyclosporine A, an immunosuppressant for the treatment of ocular inflammatory diseases, He et al. encapsulated this hydrophobic drug in PLGA microsphere. Refs. [97,98], The intravitreally injected, 50 µm sized cyclosporine A/PLGA microspheres showed a significant therapeutic effect for chronic uveitis in rabbits. Another example of particularization for retinal delivery is Visudyne^®^, a clinically approved photodynamic therapy formulation [99,100]. It is a liposome-based hydrophobic photosensitizer for the treatment of choroidal neovascularization caused by AMD. Intravenously injected liposomes can reach the retina and choroid through the abnormal choroidal blood vessels where they accumulate. Nonthermal red light triggers the release of oxygen free radicals, resulting in blockage to the vessels. Recently Shim et al. presented a novel eyedrop dosage form of a hydrophobic drug using PEG for the treatment of dry eye disease [101,102]. Catechin, a water-insoluble flavonoid exhibiting anti-oxidant and anti-inflammatory effects, was stably solubilized in PEG solution forming nano-scale particles via hydrogen bonding between the drug and PEG. In addition to solubility enhancement, particle formation can be used for a long-term/controlled drug release, drug protection against enzymatic degradation, and cytosolic delivery, which are discussed in the following sections. Although high amounts of excipients and a multi-step process are required for this system, it has a high degree of freedom of surface modifications for multifunctionality, such as targeting ligand decoration. This method also avoids any direct modification of the drug compound, which can have unknown effects on the target binding [103].

#### 3.3.2. Enhanced Retention Time

While it is clear that intravitreal injection is typically more effective than other delivery methods for retinal drug delivery, repeated injections carry a variety of risks, such as retinal detachment, uveitis, ocular hypertension, and intraocular hemorrhaging [46]. To avoid these complications, several formulation strategies have been developed to enhance the drug retention time in the vitreous and decrease the frequency of intravitreal administration.

#### 3.3.3. Lipophilization

**Figure 8:**
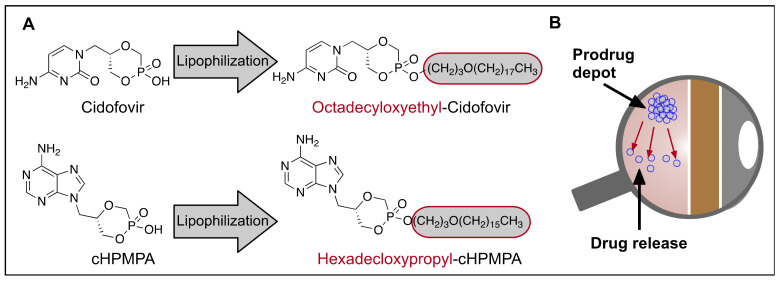
Lipid prodrugs have successfully been used to prolong the vitreal half-life of small hydrophilic compounds. (**A**) Conjugation of cyclic Cidofovir to a lipophilic chain and lipophilization of cHPMPA results in the formation of a depot after intravitreal injection which lasts significantly longer than vitreous retention times. (**B**) After injection of lipophilic prodrugs, a depot forms within the eye from which the drug is slowly released.

Although drug solubilization is the essential formulation step for practical use, precipitation of highly insoluble drugs or lipid prodrugs in the physiological condition can enhance their half-life by slowly releasing the active form of drugs. Following the intravitreal injection of a solution containing a highly insoluble drug, a local drug reservoir may form from the drug precipitating out of solution. These insoluble drugs typically undergo an initial, rapid diffusion into the surrounding tissues followed by precipitation at the injection site. Prinomastat, a matrix metalloproteinase inhibitor with poor water solubility (0.0464 mg/mL), can be temporarily dissolved in acidified water. In the study of Cheng et al., after injection, the drug slowly diffused from this precipitate, which resulted in an extended vitreous half-life of 100 h [104]. The drug concentration in the retina dropped faster than in the choroid, probably due to the fast penetration of the small and lipophilic Prinomastat from the retina towards the choroid. Therapeutic concentrations in the choroid were maintained for up to 4 weeks, suggesting that this drug formulation has a potential as a long-lasting treatment for choroidal neovascularization. Lipid prodrugs, i.e., lipid derivatized drugs, have also been designed to precipitate in the vitreous in order to achieve longer intravitreal half-lives [105,106,107]. Alkoxyalkyl prodrugs are particularly useful as their lipophilic property is able to increase their retention time and membrane permeability (Figure 8). Alkoxyalkyl chains can then be easily cleaved via cellular enzymes, resulting in release of the parent drugs. This technique has been used for the long-lasting efficacy of both retinal drugs cidofovir and 9-(S)-(3-Hydroxyl-2-Phosphonomehoxypropyl)adenine (cHPMPA), which are hydrophilic small nucleoside antiviral drugs used to treat retinal inflammation caused by cytomegalovirus (CMV) [106,108]. The vitreous half-life of cidofovir was measured to be 20 h while the octadecycloxyethyl-cidofovir prodrug containing a long lipophilic alkyl chain showed a 25-day half-life, indicating that the precipitated lipophilic drugs are cleared slower than the soluble drugs in the vitreous [109]. In the study of Tammewar et al., a crystallized depot of alkoxyalkyl cHPMPA prodrug was observed in the vitreous soon after intravitreal injection to rabbit eyes [106]. This depot remained for 18 weeks. The drug release profile can be further modulated by tuning hydrophobic moieties [110]. This precipitation strategy is not ideal for large peptide or protein drugs due to the dramatic decrease in their activity after precipitation.

#### 3.3.4. Implant

**Figure 9:**
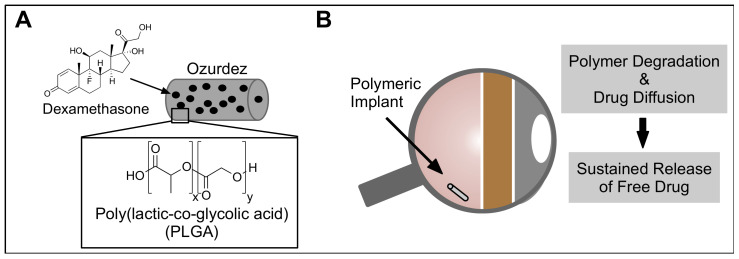
Drug encapsulation into biodegradable polymeric implant for extension of drug retention time and reduction in dosage frequency. (**A**) Ozurdez is an ocular implant consisting of dexamethasone entrapped in PLGA polymeric scaffold. (**B**) Biodegradable polymeric implant is intravitreally injected within the eye, slowly releasing free drug by polymer degradation and drug diffusion.

Another delivery method that has gained attention recently is the use of intraocular implants. Intraocular implants have been designed to release drugs in the vitreous at a slow, sustained rate over extended periods of time, greatly reducing the number of doses required. This system enables a high drug loading in the drug depot and avoids a burst release while effectively delivery drugs to the posterior chamber of the eye. These implants are typically composed of either a non-degradable implant that releases the drug over time through a permeable end-part of the implant body or a biodegradable material, such as poly(vinyl) alcohol, poly(lactic glycolic acid), and polyamide, that slowly releases the drug in the vitreous via scaffold degradation and drug diffusion. Currently, four different ocular implants are approved by the FDA to treat different ocular disorders and many more are currently in various stages of clinical trial development [111]. Corticosteroids, small lipophilic hormones that usually have a short intravitreal half-life (~ 3 h) due to their fast elimination from the vitreous have been formulated into implants [112]. Ozurdex^®^ and Iluvien^®^ are two of the most common implants and contain dexamethasone in a PLGA matrix and PVA (Poly-vinyl alcohol)/fluocinolone acetonide in a polyimide tube, respectively (Figure 9). They show an extended duration of action and provide treatment for diabetic macular edema for more than 6 months and 2–3 years, respectively [111]. Since chemical modifications are not necessary to modify the pharmacokinetics, this method is extremely attractive for small molecule and peptidomimetic delivery. Once the appropriate drug and dosage is identified, an implant can be designed and developed.

#### 3.3.5. Particlization and Encapsulation

Nano- and micro-particle formulation is another strategy to obtain prolonged residence time in the vitreous by slowly releasing free drugs from the particle scaffold. Li et al. showed the longer-term protection of retinal ganglion cell layers under ischemic injury conditions by intravitreally injected PLGA nanoparticles loaded with pigment epithelium-derived factor (PEDF) peptides compared to free form peptides [113]. Kim et al. encapsulated water-soluble integrin-antagonist peptide, C16Y, in the biodegradable polylactic acid/polylactic acid-PEG nanoparticles [114]. The intravitreally injected nanoparticles showed more efficient inhibition of choroidal neovascularization than free peptides. The authors explained that these superior effects are likely due to the enhanced pharmacokinetic properties, such as prolonged retention time of nano-sized drug depot in the vitreous and the prevention of proteolytic degradation. Xu et al. evaluated the attenuated mobility of nanoparticles with different sizes and various surface properties (positive charged, negative charged, and PEGylation) using bovine vitreous ex vivo [32]. Since the vitreous mesh is composed of highly negatively charged hyaluronic acid, positively charged particles (~ 200 nm) showed only marginal mobility due to coulombic interaction with the vitreous meshwork. Negatively charged particles and PEGylated particles showed hindered movement in the vitreous when the particle sizes were larger than 500 nm and 1000 nm, respectively. This study indicated that large particles with sizes of 1000 nm or above can be used for the sustained release of retinal drugs as they would likely have slow vitreous clearance rates. Self-assembled nanoparticle formation of small peptides or their derivatives can be an alternative strategy without using polymeric or lipidic additives [115]. By modulating peptide side chains or conjugating polymers, peptide drugs can be self-assembled via intermolecular interactions, e.g., hydrophobic, hydrogen, and π-π interactions. Unlike the solid ocular implants administered either by surgical insertion or by specified injector equipped with a large sized needle (e.g., 25-gauge), this particle platform could be applied through a less-invasive intravitreal injection with a needle size range of 27- to 30-gauge [33,116]. However, the manufacturing of nanoparticles with homogeneous size and drug loading is crucial to avoid undesired drug elimination and release fluctuation.

#### 3.3.6. Binding to Intraocular Molecules

Melanin, a polyanionic pigment macromolecule derived from tyrosine oxidation, has been known to bind with various drugs and affect their retention time [117]. The choroid and RPE contain a high level of intracellular melanin formed into melanosomes. Once drugs penetrate these cells, they can bind to melanin and form a reservoir, which releases free drug slowly. For this to happen, drug/melanin binding affinity and intracellular permeability of the drug are the critical factors determining the pharmacokinetics of retinal drugs [117]. For example, chloroquine, which exhibits a high binding affinity, shows an extremely long retention time (for months) in pigmented eye tissues [118]. Narayan et al. showed the superior retention of a lipophilic compound within the Bruch’s membrane and choroid in bovine and porcine eyes, probably due to the melanin binding. Robbie et al. demonstrated that melanin binding of lipophilic pazopanib and GW771806 enables drug retention within the uveal tract of rat eyes for several weeks after a single oral dose while most drugs in albino rat eyes were eliminated within 3 days [119]. The ocular half-life for pazopanib and GW771806 after 100 mg/kg oral dosing was measured to be approximately 18 days. A single oral dosing of pazopanib resulted in the significant therapeutic effect of Choroidal Neovascularization (CNV). As an alternative to oral dosing, to minimize systemic toxicity, pazopanib and GW771806 were administered via topical instillation and showed efficient accumulation in the choroid and RPE. However, only GW771806 showed significant alleviation of CNV, presumably due to the higher depot level of GW771806 compared to pazopanib. This study highlights that melanin/drug binding not only increases drug half-life by forming a drug reservoir, but also facilitates the efficient drug delivery to the posterior segment via non-invasive topical or oral administration routes. Based on these studies, introduction of a melanin binding moiety to fast-eliminating-drugs can be an alternative strategy to increase ocular retention time and prolong therapeutic effects. Besides melanin-binding, combination of a hyaluronan-binding peptide with an anti-VEGF protein drug resulted in longer retention time in the treatment of neovascular retinal disease. These fusion proteins showed a prolonged retention time in vitreous of rabbits and monkeys and their therapeutic effects lasted 3-4-fold longer than those of unmodified proteins. These hyaluronan-binding peptides could readily be appended to peptidomimetics to generate prodrugs with extended vitreous retention.

#### 3.3.7. PEGylation

Conjugation of large molecular weight PEG units to a drug results in a higher hydrodynamic volume compared to the free drug, which can prolong the residence time. For example, Pegaptanib (Macugen), an anti-VEGF RNA aptamer including a large molecular weight (40 kDa) of PEG for the treatment of neovascular AMD, showed prolonged tissue retention due to increased molecular size. Another example is Pegcetacoplan, a PEGylated complement C3 inhibitor cyclic peptide wherein a large molecular weight PEG moiety (40 kDa) was introduced for long residence time [120]. Recent results of its clinical trial phase II showed that the intravitreal injections of 15 mg Pegcetacoplan monthly or every other month for one year resulted in a significant therapeutic effect with AMD patients. Similarly, Machinaga et al. conjugated high molecular weight 4-arm PEG (40 kDa) to small drugs via a self-cleaving linker, exhibiting a long cleavage half-life [121]. Intravitreal injection of these PEGylated drugs showed a long-acting effect within rabbit vitreous due to the increased retention time and slow release of the free drugs.

#### 3.3.8. Increased Enzymatic Resistance

Since the vitreous contains a high level of proteolytic enzymes, such as matrix metalloproteinase and serine/cysteine protease, peptides/peptidomimetics need to be protected against enzymatic attack. Since the concentration of some proteolytic enzymes levels in the vitreous is age- and disease-related, formulation of retinal drugs needs to be optimized to exhibit the corresponding enzyme-resistance [122,123]. In the case of peptide drugs, as mentioned above, D-form peptides- or peptoid-type peptidomimetics are a most common strategy to increase enzymatic resistance [124]. In addition, chemical modifications at the N and C terminus, e.g., C-terminal amidation or N-terminal acetylation, make the peptide more difficult for proteolytic enzymes to recognized [125,126]. PEGylation can shield biopharmaceutics, such as peptides, proteins, and genes, resulting in a lower propensity to undergo enzymatic cleavage [127]. For examples, PEGylated calcitonin and glucagon-like peptide-1 (GLP-1) resulted in significantly increased stability against plasma and intestinal peptidase attack, respectively [128,129]. However, there have been no studies to evaluate proteolytic resistance of PEGylated peptide drugs in the vitreous.

Besides proteolytic enzymes, eye tissues contain high amounts of metabolic enzymes, including cytochrome P450 reductases and lysosomal enzymes, which are responsible for eye protection and maintenance of homeostasis [130,131,132]. Cytochrome P450 is known to play a pivotal role in metabolizing steroids, fatty acids, and xenobiotic ocular drugs. Encapsulation of retinal drugs in nanoparticles or an implant matrix can attenuate drug degradation during delivery by minimizing the enzyme access [133].

#### 3.3.9. Tissue Specific Delivery

Tissue-selective drug delivery can be achieved by modifying drugs or their carriers to exhibit superior membrane permeation at the target tissue via tissue-specific transporters or receptors. This target tissue-specific delivery can prevent drug accumulation in the undesired neighboring tissues, which can potentially cause toxicity, as well as increase the local drug concentration allowing one to reduce the administration dose.

**Figure 10:**
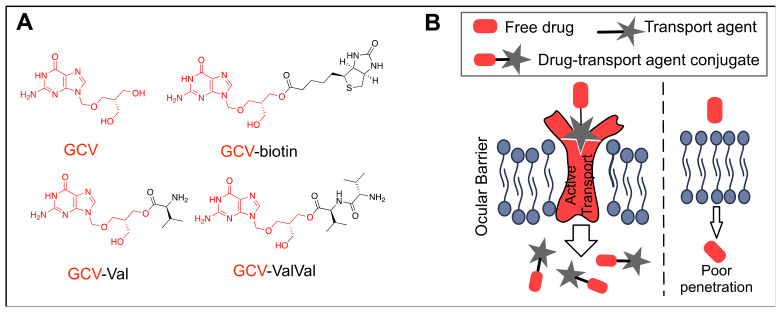
Drug conjugation with transport agents engaging in enhanced uptake via membrane transporters. (**A**) Ganciclovir (GCV), a polar and hydrophilic antiviral drug, is conjugated with biotin, di-valine (ValVal), mono-valine (Val) for active uptake. (**B**) Drug-transport agents conjugation enhances drug permeability by penetration through active transporters.

Transporters play a pivotal role in traversing ions, small molecules, and peptides across the lipid plasma membrane. While most lipophilic small molecules can penetrate through the cell membranes passively, hydrophilic drugs can append small linkers recognized by specific active transport systems to aid in permeation. Ganciclovir (GCV), an antiviral drug to treat CMV infection, has poor membrane permeability due to its hydrophilicity. Apt et al. showed the increased retinal uptake after intravitreal injection by appending peptide subunits to the parent GCV molecule, aimed at targeting peptide transporters (Figure 10) [134]. The authors found that administration of Val-GCV and Val-Val-GCV prodrugs significantly reduced the retinal uptake of glycylsarcosine (Gly-Sar, a model dipeptide substrate for assaying transepithelial transport of small molecules by peptide transporters), suggesting that Val-GCV and Val-Val-GCV prodrugs may be substrates of the retinal peptide transport system. This hypothesis was corroborated by the pharmacokinetic studies which revealed that retinal GCV concentrations 5 h after the prodrugs administration were almost twice that compared to the parent GCV. Another example is the use of biotinylated GCV for the enhanced transmembrane transportation through the RPE [135]. Janoria et al. demonstrated that intravitreally injected biotin-GCV can be transported to the retina-choroid via the Sodium/Multivitamin Transporter (SMVT) system expressed in the RPE. However, the total accumulation of biotin-GCV in the retina-choroid was similar to the accumulation of GCV, suggesting that passive transportation may still play a critical role in the overall drug concentration in the retina-choroid.

Receptor-mediated drug delivery is also a promising strategy to enhance drug permeation at the target tissue with a high selectivity and efficiency. Receptors are expressed differently depending on cell types, and their expression levels can be modified under pathological conditions [136]. Combined with high feasibility of local administration to the eyes, this receptor-mediated delivery is beneficial for ocular tissue-specific delivery with a minimal systemic exposure of drugs. Since receptor/ligand binding can often enhance the receptor-mediated endocytosis, various ligands have been introduced to drugs not only for tissue-specific delivery with minimal toxicity, but also for the intracellular delivery of membrane impermeable drugs such as macromolecular biopharmaceutics. Table 1 presents the diverse receptors expressing in the eyes and the corresponding ligands for ocular tissue-specific drug delivery. Receptor specific ligand molecules can be conjugated to the drug backbone or drug carrier.

Transferrin receptor- and integrin-specific ligands (e.g., transferrin and RGD, respectively) have been used in a retina-specific drug delivery platform, especially for neovascularization treatment. Zhu et al. introduced the transferrin receptor-specific monoclonal antibody on the surface of transgene containing liposomes [138]. After intravenous injection of these liposomes in mice, transgenes were expressed throughout the RPE and other structures of the eye, indicating that this formulation can be used for eye-targeting gene therapy via non-invasive treatment. Singh et al. formulated anti-VEGF interceptor genes containing PLGA nanoparticles decorated with RGD peptide, transferrin, or both ligand molecules [139]. Intravenously injected particles were accumulated in the retina via RGD- and transferrin-mediated transcytosis. Furthermore, the particles in the retina were intracellularly delivered into the RPE cells to produce anti-VEGF proteins, resulting in significant CNV therapeutic effects compared to the particles without ligands. Recently, Chandola et al. demonstrated an RPE specific drug delivery platform by using the CD44 receptor overexpressed in the RPE under oxidative stress, which potentially causes AMD and vitreoretinopathy [137]. CD44-specific RNA aptamer-FITC conjugates were efficiently taken up by the RPE overexpressing CD44 receptors via a receptor-mediated endocytosis pathway.

Apart from retina-specific delivery, Henning et al. demonstrated the RGD receptor-mediated intracellular delivery of quantum dots in the trabecular meshwork [140]. As a ligand targeting corneal membrane, lactoferrin, an iron-binding protein of the transferrin family, was used. Higuchi et al. introduced selenium, which exhibits an anti-oxidative effect, at the iron-binding site in lactoferrin and this selenium-bound lactoferrin was administered topically to a dry eye disease rat model [141,142]. Selenium-lactoferrin showed the efficient uptake into corneal epithelium via the lactoferrin receptor, resulting in a superior improvement of dry eye compared with control selenium compounds. Although there have been only a few studies demonstrating the ocular tissue-specific drug delivery via targeting ligands, various receptors overexpressed in eye tissues have a potential for the tissue-selective delivery of ocular drugs. For example, the expression levels of receptors for EGF, [143] mannose, [144] and folate [145] are upregulated in the cornea, RPE, and RPE/retina, respectively. These receptors, which are also overexpressed in cancer cells, have been extensively used for tumor-specific anticancer drug delivery and exhibit great potential for ocular tissue-targeting drug delivery [146,147,148].

#### 3.3.10. Enhanced Membrane Permeability

Among the various ocular tissues, the cornea, non-pigmented epithelium of ciliary process, retinal capillary endothelium, and RPE have tight junctions, which limit the paracellular transport of drug molecules. To penetrate the tissues containing tight junctions, drug molecules need to be transported via a transcellular route, i.e., traversing through the lipid cell membrane. This is why most large and hydrophilic drugs, exhibiting a low cell membrane permeability, are injected into the vitreous to bypass the tight junction tissues to reach the retina. For efficient retinal delivery of these drugs via topical and systemic administration, penetration enhancers, temporarily destabilizing membranes, or loosening tight junctions, can be co-administered with or conjugated to drugs.

Cell penetrating peptides (CPPs) are short peptides facilitating intracellular uptake via endocytosis or direct translocation. CPPs can deliver various types of drug cargos, such as small drugs, peptide, protein, and genes, by co-treatment, complexation, or conjugation form. Due to their excellent membrane modulation actions, CPPs have been used as a penetration enhancer to overcome drug absorption barriers such as epithelial tight junctions in the intestine, skin, eye, etc. [149,150,151]. Various CPPs, such as TAT (Transactivator of Transcription), polyarginine, and penetratin, demonstrated their efficient membrane permeability by delivering topically administered drugs to the posterior segment via either direct access from the aqueous humor after penetration of the cornea epithelium or RPE penetration after a conjunctival-sclera pathway.

Topically delivered TAT-fibroblast growth factor (FGF) conjugates reached the rat retina and remained for 8 h [152,153]. Anti-angiogenesis protein, endostatin, was expressed as a TAT fusion protein and this endostatin-TAT (22 kDa) was administered topically to mouse eyes [154]. The protein was efficiently delivered to the retina, resulting in the inhibition of choroidal neovascularization. Surface decorations of PLGA nanoparticles with TAT facilitates the efficient delivery of particles to the posterior segment via topical administration [155].

Polyarginine (R6) was conjugated to anti-VEGF monoclonal antibody agents, i.e., bevacizumab and ranitizumab [156,157]. These conjugates were topically administered in rats and the drugs were observed in the aqueous humor and the vitreous/retina, indicating that R6-antibody drugs can penetrate the corneal epithelium and RPE, respectively. Daily treatment of these conjugates in a CNV mouse model via topical instillations showed a significant therapeutic effect, indicating that the CPP-mediated permeability enhancement enabled the efficient retinal delivery of macromolecule drugs via non-invasive topical administrations.

Penetratin has been used for the retinal delivery of macromolecules, especially nanoparticles. Complexes of penetratin/Red Fluorescent Protein expressing plasmid (pRFP) with and without poly(amidoamine) (PAMAM) were administered via topical instillation to the rat conjunctival sac [158]. After consecutive administrations every 8 h for 3 days, both penetratin/pRFP and penetratin/PAMAM/pRFP expressed fluorescent protein in the retina. The addition of PAMAM for particle condensation enhanced the gene delivery efficiency. Similarly, hyaluronic acid/PAMAM complexes with or without penetratin were used for the retinal delivery of antisense oligonucleotide [159]. Fluorescence images of the retina showed that the retinal accumulation of topically instilled hyaluronic acid/PAMAM/antisense oligonucleotide complexes was greatly enhanced by penetratin incorporation. Suda et al. demonstrated a CPP-presenting high-density lipoprotein (HDL) mutant particle as an efficient retinal drug carrier [160]. This HDL particle is composed of apolipoprotein A1 fused with penetratin and phospholipids with a size of 15 nm. Topical administration of the anti-angiogenesis drug pazopanib carried by these penetratin-presenting HDL particles showed significant therapeutic effects in a murine AMD model. Jiang et al. demonstrated the increase in membrane penetration capacity of penetratin in eyes by increasing its hydrophobicity [161]. Ocular distribution of topically administered fluorophore labelled penetratin and hydrophobic penetratin derivatives, wherein basic amino acid residues of original peptide are replaced with tryptophan, was compared in vivo. The result showed that hydrophobic penetratin derivatives exhibit higher fluorescent accumulation levels in the cornea and retina than penetratin. Although this study provided a promising strategy in CPP sequence modification for retinal drug delivery, the overall in vivo pharmacokinetic profiles of modified CPP after drug incorporation needs confirmation.

A Peptide for Ocular Delivery, POD, was designed to increase the ocular penetration capacity of conjugated drugs [162]. Subretinal and intravitreal injection of POD conjugates with various drug cargos (e.g., lissamine, quantum dot, and GFP [Green Fluorescent Protein]) showed the rapid localization in RPE/photoreceptor cells and ganglion cells, respectively [163]. In the case of topical administration, the membrane permeability of POD conjugates and the consequent ocular distribution varied depending on the cargo types. For example, topical administration of POD-lissamine resulted in the significant dye accumulation in the cornea epithelium, sclera/choroid region, and optic nerve, while only corneal epithelium localization was presented after topical application of POD-GFP. POD was introduced to the nanoparticle system incorporating various drugs, i.e., small compound and siRNA, for their corneal epithelium delivery via topical instillation [164,165]. Although POD enhances drug permeability, this CPP is likely not a good candidate for retinal delivery via topical administration due to lack of its RPE penetration capacity.

Considering the fact that drug delivery efficiency of CPP can be dramatically changed by CPP type, drug cargo, formulation method, and administration route, activity screening of a wide range of CPP libraries with different types of drugs can provide valuable information for use of CPP as a retinal drug carrier. Liu et al. compared the penetration efficiency of various fluorophore labelled CPPs, such as penetratin, polyarginine (R8), TAT, and protamine [166]. After topically administered into rat eyes, penetratin showed the most efficient peptide distribution in both the anterior and posterior chamber compared to other CPPs. Penetratin was detected in the corneal epithelium and retina for 6 h. Pescina et al. examined the different corneal penetration capacity of CPPs in ex vivo bovine eyes using fluorophore labelled penetratin, PEP-1 and PEP-1 derivatives [167,168,169]. Penetratin showed a highly efficient transcellular transport while PEP-1 and PEP-1 derivatives were displayed in the intercellular space, suggesting that penetratin and PEP-1/PEP-1 derivatives can be selectively used for intracellular and paracellular drug targeting, respectively. Recently Chen et al. discovered a novel dodecapeptide, CC12, exhibiting excellent ocular permeability through the in vivo-directed phage display technology [170]. This CPP was applied for the retinal delivery of KV11, an anti-angiogenetic peptide to inhibit retinal neovascularization, in a conjugate form. A comparative study in vivo showed that the ocular permeability of CC12 and its KV11 conjugates were similar to penetratin. Noninvasive (via conjunctival sac instillation) or minimally invasive (via retrobulbar injection) administration of CC12-KV11 showed a significant therapeutic effect in an oxygen-induced retinopathy mouse model.

Besides CPPs, surfactants, amphiphilic compounds, bile acids, crown ethers, and calcium chelating, agents have been used for enhanced membrane permeation of ocular drugs [171,172,173,174]. Cyclodextrin (CD) also helps drugs to penetrate the cornea by interacting with epithelium cholesterol or other lipids [175]. A recent review from Moiseev et al. describes various penetrating enhancers for ocular drug delivery and their mode of actions [176].

## 4. Perspectives—Design of Peptidomimetics for Proteases

Peptidomimetic drugs have the potential to treat a variety of ocular diseases due to their target specificity. Using proteomics and genomics studies from patients, many mis-regulated proteases have been shown to contribute to a variety of ocular disorders and are potential targets for drug development. After discovery and validation of a protein associated with disease, a suitable biochemical assay is needed to test the effect of the drug on the function of the protease. This can readily be achieved for proteases using fluorescently labeled peptide substrates. This assay can be used to screen potential inhibitors to identify hits and lead-like compounds. During this process, cellular and animal models can be developed for more advanced testing. Once a series of lead-like compounds are identified, pharmacokinetic data are gathered, and these compounds can be tested in the cellular assays. After initial toxicity studies, these compounds are tested in animals and the in vivo pharmacokinetic data such as ADME (Absorption, Distribution, Metabolism, and Excretion) are collected. At this stage, only the top compound with the appropriate efficacy and pharmacokinetic properties is moved forward for testing in larger animals.

Small peptidomimetics can be beneficial in formulation due to their low structural complexity while large peptidomimetics, exhibiting more protein-like characteristics, need to be formulated with a consideration for activity loss from to structural modifications. For intracellular retinal drugs, targeting cytosolic molecules or genomes, additional pharmacokinetic factors such as intracellular uptake, endosome escape, and nucleus targeting need to be considered.

Once a sufficiently potent and selective drug candidate is identified, different formulation strategies can be attempted to optimize ocular delivery. To overcome the intrinsic pharmacokinetic drawbacks and achieve the valid biological effect of peptidomimetics, the appropriate administration routes and formulation methods need to be selected. Peptidomimetic drug development to target the retina can be challenging due to limited drug access, the unique ocular anatomy and a variety of unique ocular barriers. Various drug formulation strategies presented in in this review (Table 2) can be adapted for the retinal delivery of peptidomimetics. A variety of administration routes exist with their own unique advantages and disadvantages. For example, intraocular injection is most effective for treating the posterior segment diseases while topical administration often enables the treatment of anterior segment diseases. Therefore, when developing a drug, the method of administration can be determined accordingly based on the targeted region. Although intravitreal injection results in the highest bioavailability of retinal drugs, and most clinically available retinal drugs are intravitreally injected, the non-invasive topical/oral administrations routes may warrant consideration since patients can self-administer treatment. However, topical and oral delivery is highly challenging due to various biological and physical barriers that must be overcome before reaching the posterior segments.

## 5. Conclusions

Peptidomimetics are a promising class of ocular drugs due to their selective biological function and molecular stability. Although intensive in vivo and in vitro drug screening enables the selection of potent peptidomimetic drugs, their therapeutic effects in the eye are varied due to their different pharmacokinetic profiles, especially for the retina due to its various physical barriers. The selection of appropriate formulation methods and administration routes enables one to overcome the pharmacokinetic drawbacks. We have discussed several delivery routes and formulation tools for addressing the typically short half-lives and poor membrane permeability of retinal drugs. Many successful examples have demonstrated the various strategies that can be used to enhance the pharmacokinetics of small or large retinal drugs, and these techniques should extend to peptidomimetics. A variety of pharmacokinetic modifications in conjunction with a plethora of ocular administration techniques make peptidomimetics a promising class of compounds for the treatment of retinal disorders.

## Figures and Tables

**Table 1:** Various receptor-specific ligands to target specific ocular tissues.

Receptor	Ligand	Delivery System	Target Tissue
Transferrin receptor	Transferrin (protein)	Liposome as gene delivery carrier [137]	Multiple areas (conjunctiva, iris, ciliary body, cornea, RPE) via intravitreal injection (in vivo)
		PLGA nanoparticle as gene delivery carrier [138]	RPE via intravitreal injection (in vivo)
Integrin	RGD (peptide)	Quantum dot [139]	Trabecular meshwork (in vivo)
		PLGA nanoparticle as gene delivery carrier [140]	RPE via intravitreal injection
Lactoferrin receptor	Lactoferrin (protein)	Selenium/lactoferrin complex [141,142]	Cornea via topical instillation (in vivo, rat)
CD44 receptor	CD44 aptamer (RNA)	FITC conjugate [137]	RPE under oxidative stress (in vitro)

**Table 2:** Additives and formulation methods to achieve essential pharmacokinetic properties for the efficient retinal delivery of peptidomimetic drugs.

Purpose	Additives/Formulations	Outcomes
Solubility improvement	Hydrotropes Cyclodextrin (CD) Salt formation PEGylation Particle formation	Enhanced injectability Increased bioavailability
Enhanced retention	Precipitation Lipid prodrug Implant Melanin binding Particle formation PEGylation Conjugation of hyaluronic acid-binding moiety	Long-lasting drug effect Reduced injection time
Increased enzymatic resistance	Peptidomimetic form (D-form, peptoid) Chemical modifications at terminus PEGylation Particle formation	Long-lasting drug effect
Tissue specificity	Transporter recognizing molecule Receptor specific ligand	Decrease in injection dose Reduced toxicity
Enhanced permeability	CPPs, surfactants, bile acids, amphiphilic compounds, crown ethers, and calcium chelating agents, cyclodextrin	Increase in drug bioavailability Intracellular delivery
